# Astrocytic Hevin/SPARCL‐1 Regulates Cognitive Decline in Pathological and Normal Brain Aging

**DOI:** 10.1111/acel.14493

**Published:** 2025-02-12

**Authors:** Felipe Cabral‐Miranda, Ana Paula Bergamo Araujo, Danilo Bilches Medinas, Flávia Carvalho Alcantara Gomes

**Affiliations:** ^1^ Institute of Biomedical Sciences Universidade Federal do Rio de Janeiro Rio de Janeiro Brazil; ^2^ Department of Biochemistry, Institute of Chemistry University of São Paulo São Paulo Brazil

**Keywords:** aging, Alzheimer's disease, astrocyte, Hevin, SPARCL‐1

## Abstract

Dementia, characterized by loss of cognitive abilities in the elderly, poses a significant global health challenge. This study explores the role of astrocytes, one of most representative glial cells in the brain, in mitigating cognitive decline. Specifically, we investigated the impact of Hevin (also known as SPARC‐like1/SPARCL‐1), a secreted glycoprotein, on cognitive decline in both normal and pathological brain aging. By using adeno‐associated viruses, we overexpressed Hevin in hippocampal astrocytes of middle‐aged APP/PSEN mice, an established Alzheimer's disease (AD) model. Results demonstrated that Hevin overexpression attenuates cognitive decline, as evidenced by cognitive tests, increased pre‐ and postsynaptic markers colocalization, and altered expression of synaptic mediators, as revealed by proteomic profiling. Importantly, Hevin overexpression did not influence the deposition of Aβ plaques in the hippocampus, a hallmark of AD pathology. Furthermore, the study extended its findings to middle‐aged wild‐type animals, revealing improved cognitive performance following astrocytic Hevin overexpression. In conclusion, our results propose astrocytic Hevin as a potential therapeutic target for age‐associated cognitive decline.

## Graphical abstract

Hevin/Sparcl‐1 is a synaptogenic protein secreted by astrocytes. We used adeno‐associated virus (AAV) to increase its expression in hippocampal astrocytes of APP/PSEN1 mice, an animal model for Alzheimer's disease (AD). This procedure improved cognitive performance both in the AD model and in middle‐aged wild‐type animals. Proteomics profiling indicated that a cluster of synaptic molecules were impacted and importantly, there was no effect in the deposition of Aβ plaques in the brains of AD animals. We propose astrocytic Hevin as a potential therapeutic target for age‐associated cognitive decline.

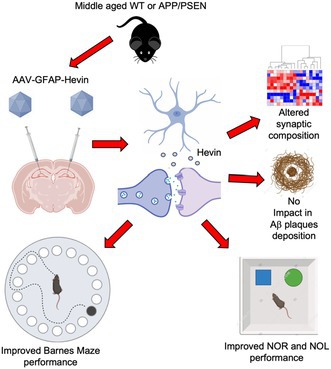

## 1 Introduction

Dementia is characterized by a significant loss of cognitive ability that hinders daily functioning with increased incidence in the elderly population (Livingston et al. 2020), posing a significant global health challenge. It affects around 50 million people worldwide, and this number is anticipated to rise to 152 million by 2050, with a disproportionate impact on low‐income and middle‐income countries (Livingston et al. 2020). The condition brings a considerable burden on individuals, families, and the economy, with estimated global costs reaching approximately US$1 trillion annually. Alzheimer's disease (AD), the most prevalent dementia worldwide, is marked by neuronal and synaptic loss, associated with cognitive decline, along with the accumulation of amyloid‐β (Aβ) plaques and phosphorylated tau in neurofibrillary tangles (Tzioras et al. 2023). However, more recent evidence suggests that soluble forms of Aβ and tau, rather than the traditionally emphasized plaques and tangles, play a direct role in synaptic toxicity and cognitive deficits (Kopeikina, Hyman, and Spires‐Jones 2012; Ferreira et al. 2015).

Astrocytes play a crucial role in maintaining brain homeostasis by shaping synapses, influencing their formation, maturation, maintenance, pruning, and impacting neuronal plasticity (Allen and Eroglu 2017; Matias, Morgado, and Gomes 2019; Lawal, Ulloa Severino, and Eroglu 2022). Astrocytes also offer intrinsic neuroprotection to neurons, and their neuroprotective features are being explored as potential therapeutic interventions in conditions such as stroke and neurodegeneration including AD (Rodríguez‐Giraldo et al. 2022). Although animal studies have provided insights into the role of astrocytes and microglia in synaptic pruning, the understanding of this process in humans during aging and AD remains limited (Tzioras et al. 2023). Astrocytes release various synapse‐modifying molecules (Diniz et al. 2012, 2014; Buosi et al. 2018; Matias, Morgado, and Gomes 2019; Tan, Burrus Lane, and Eroglu 2021), including members of the secreted protein acidic and rich in cysteine (SPARC) family proteins such as Hevin (also known as SPARC‐like1/SPARCL1) and SPARC.

Hevin, a secreted glycoprotein, has been reported to induce the development of structurally formed but functionally silent synapses, while its antagonist, SPARC, inhibits Hevin synaptogenic effects (Kucukdereli et al. 2011). Those findings suggest that Hevin acts as a positive regulator, while SPARC functions as a negative regulator of synapse formation and the relative levels of these astrocyte secreted factors may play a crucial role in shaping the formation, maturation, and plasticity of synapses in vivo. In those lines, prior research has demonstrated that Hevin plays a key role in orchestrating the establishment and fine‐tuning of thalamocortical glutamatergic synapses, which is achieved by acting as a bridge between non‐compatible presynaptic neurexin‐1 alpha (Nrx1α) and postsynaptic neuroligin‐1B (NL1B) during the developmental stages of the mouse visual cortex (Singh et al. 2016). Furthermore, earlier investigations have provided evidence that SPARC exhibits high expression levels in the brain affected by AD and co‐localizes with Aβ protein deposits, while it has been proposed that Hevin may undergo downregulation in the diseased state (Singh et al. 2016). Recently, mutations in *SPARCL1* have been identified as accelerators of symptom onset in AD in addition to exerting influence on brain structure and function during the aging process, suggesting that Hevin may pose as a potential regulator of cognitive decline during aging (Seddighi et al. 2017). Importantly, Hevin has been pointed out as a candidate factor that reverts age‐associated cognitive decline following administration of blood from young to aged animals (Gan and Südhof 2019).

Overall, compelling evidence suggests that astrocytic Hevin may exert a central role in brain aging and in the emergence of AD, but this hypothesis has not been functionally evaluated so far. Here, we investigated the functional role of astrocytic Hevin in regulating cognitive decline in both pathological and normal brain aging using adeno‐associated virus to drive Hevin overexpression in hippocampal astrocytes of middle‐aged APP/PSEN mice, a well‐established animal model that recapitulates many pathological features of AD (Van Dam and De Deyn 2011). We found that Hevin overexpression attenuated cognitive decline as measured by distinct cognitive tests, in addition to increasing the colocalization of pre‐ and postsynaptic markers and impacting the expression of synaptic mediators as indicated by proteomic profiling. Conversely, Hevin overexpression did not influence the deposition of Aβ plaques in the hippocampus of APP/PSEN mice. Remarkably, our findings were extended to middle‐aged wild‐type (WT) animals, which also displayed less cognitive decline following AAV‐GFAP‐Hevin treatment. In conjunction, our findings sustain that astrocytic Hevin could delay cognitive impairment observed both in normal and pathological brain aging by impacting the composition of hippocampal synapses without affecting the deposition of Aβ plaques. Those results sustain the hypothesis that glial‐based therapies directed to other aspects of brain pathology both in AD and overall cognitive decline may arise as potentially more prominent interventions to halt age‐associated cognitive impairment.

## 2 Results

### 2.1 Hevin Is Decreased in AD Patient Astrocytes and Middle‐Aged APP/PSEN Animals

**FIGURE 1:**
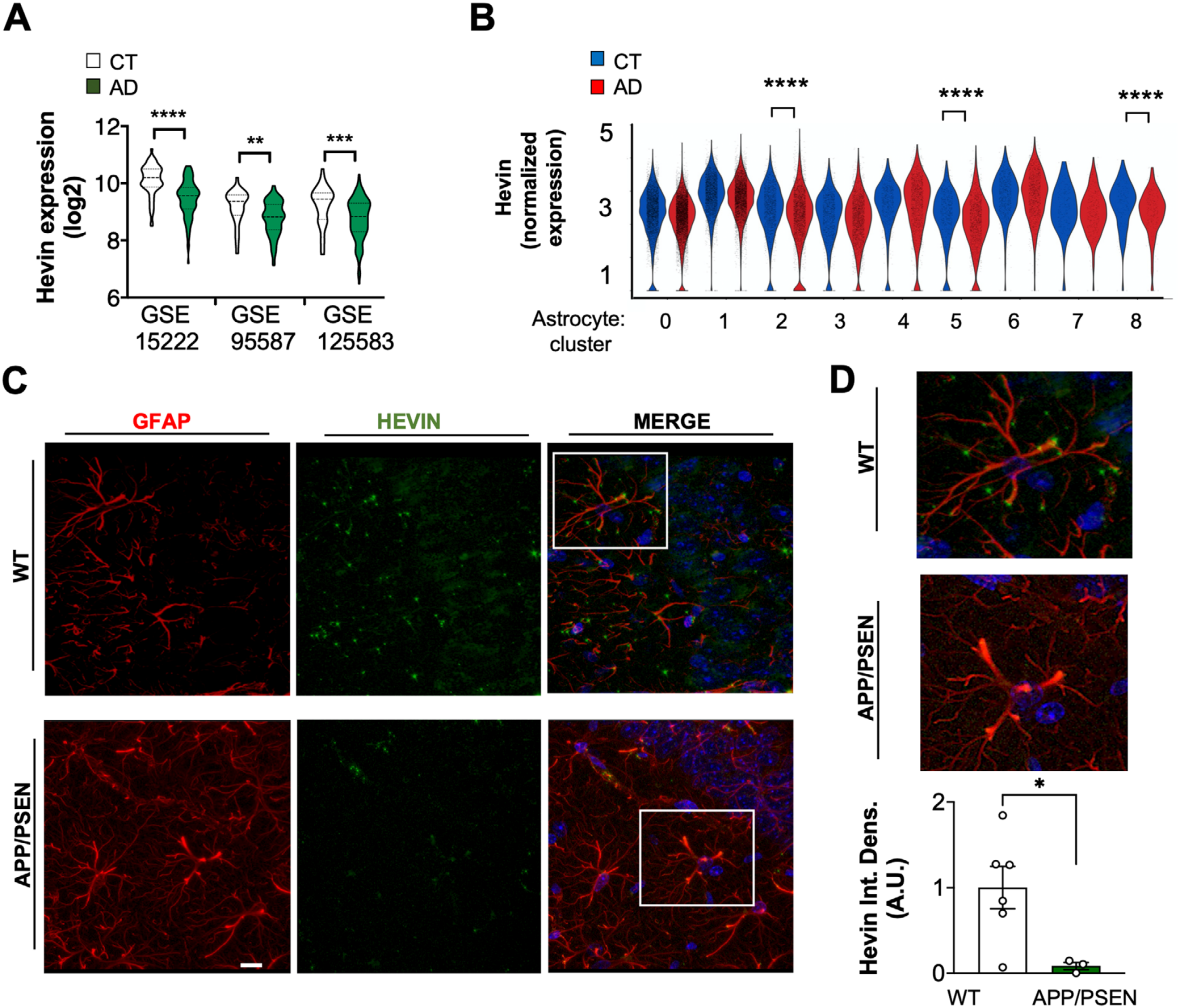
Hevin levels are decreased in astrocytes of AD patients and animal models. (A) RNAseq readings of Hevin in human datasets (GSE15222, GE95587, and GSE125583) comparing brain tissue of AD patients and controls (Kolmogorov–Smirnov test, ****p < 0.001; **p < 0.01; ***p < 0.005). AD = Alzheimer's disease patients; CT = controls. (B) Hevin expression in astrocytes of AD patients or controls as inferred by Single cell RNAseq performed in Sadick et al. (2022). Normalized expression in 8 astrocytes clusters identified in AD. Wilcoxon rank‐sum test, thresholds: Average log_2_‐fold change ±0.25, *p* < 0.05; Adjusted *p* for disease comparisons: ****p < 0.001. (C) Immunofluorescence for Hevin (green) and GFAP (red) displaying *stratum radiatum* of middle‐aged WT or APP/PSEN animals. DAPI was used to image nuclei (blue). 40× magnification, scale bar = 10 μm. (D) Magnification of images in (C) and quantification of the integrated density of the immunolabeling for Hevin. *N* = 5, 3. Unpaired Student's *t* test, ^*^ *p* < 0.05.

To validate earlier observations indicating reduced Hevin expression in the brain tissue of AD patients (Strunz et al. 2019), we conducted a comprehensive analysis of various human RNAseq datasets. These datasets compared AD patients with age‐matched healthy controls, allowing us to statistically confirm the decreased expression of Hevin in AD patients (Figure 1A). Importantly, studies employing single cell RNAseq of AD samples identified that Hevin was consistently decreased in at least 3 distinct astrocyte subpopulations associated with the disease (Figure 1B).

To further elucidate the role of Hevin in the context of AD pathogenesis, we employed the APP/PSEN mouse model, known for replicating key pathological features of AD, including Aβ plaque deposition and astrogliosis (Van Dam and De Deyn 2011). Immunofluorescence analysis of hippocampal slices from 12‐month‐old APP/PSEN mice revealed a significant reduction in Hevin immunolabeling intensity in astrocytes compared to age‐matched WT animals (*p* < 0.05; Figure 1C,D).

These findings collectively indicate a similar profile of Hevin content in astrocytes of AD animal model and patients.

### 2.2 Hevin Overexpression in Hippocampal Astrocytes Abrogates Cognitive Dysfunction Both in Normal and Pathological Aging

**FIGURE 2:**
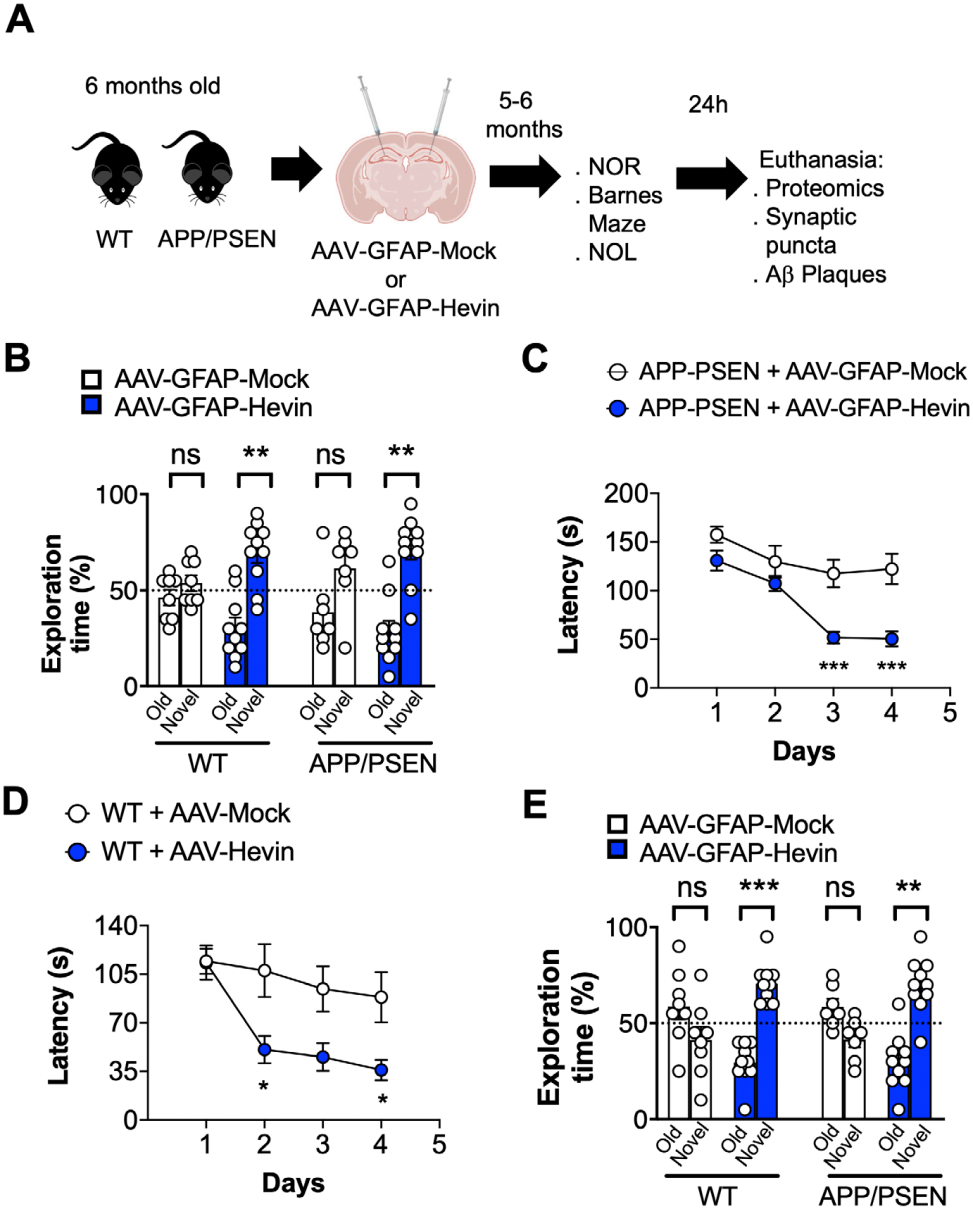
Gene delivery of Hevin in astrocytes through AAV prevents cognitive decline in middle‐aged WT and APP/PSEN animals following 6 months of treatment. (A) Workflow displaying genotypes, stereotaxic surgeries for AAV injection, behavioral tests, and end point analysis for AAV treatment for 6 months. NOL = new object location; NOR = new object recognition. (B) Percentage of exploration time of old or novel objects in the NOR task following 24 h of the training day. Middle‐aged WT or APP/PSEN animals treated with AAV‐GFAP‐Mock or AAV‐GFAP‐Hevin for 6 months were evaluated in the test. **p < 0.01, Paired Student's *t* test; Treatment Effect: *p* < 0.05, Two‐way ANOVA. (C) Primary latencies to reach the target hole during 4 consecutive days in the Barnes Maze test. Middle‐aged APP/PSEN animals treated with AAV‐GFAP‐Mock or AAV‐GFAP‐Hevin for 6 months were evaluated in the test. ***p < 0.005, Multiple *t* test. (D) Primary latencies to reach the target hole during 4 consecutive days in the Barnes Maze test. Middle‐aged WT animals treated with AAV‐GFAP‐Mock or AAV‐GFAP‐Hevin for 6 months were evaluated in the test. *p < 0.05, Multiple *t* test. (E) Percentage of exploration time of old or novel located objects in the NOL task following 24 h of the training day. Middle‐aged WT or APP/PSEN animals treated with AAV‐GFAP‐GFP or AAV‐GFAP‐Hevin for 6 months were evaluated in the test. **p < 0.01; ***p < 0.005, Treatment Effect: *p* < 0.0001, Two‐way ANOVA; Tukey's multiple comparisons.

Our findings led us to question whether manipulating Hevin expression in hippocampal astrocytes would impact the physiopathology of AD. To evaluate this hypothesis, we have performed single bilateral stereotaxic injections in the hippocampus of 6 months old APP/PSEN animals to deliver an adeno‐associated virus designed to overexpress Hevin directly to astrocytes under the control of the GFAP promoter (AAV‐m‐SPARCL‐1/AAV‐272935, Vector Biolabs), hereafter referred as to AAV‐GFAP‐Hevin. AAV constructs display a green fluorescent protein (GFP) tag to access gene distribution following the surgeries. Control littermates were injected with an AAV expressing only GFP, hereafter referred to as AAV‐GFAP‐Mock. Such long‐term regimen was implemented both for APP/PSEN and WT littermates for each viral construct (Figure 2A) in order to induce Hevin overexpression since youth and study its effects in middle age. Our results confirmed transgene distribution in hippocampal astrocytes as indicated by GFP immunofluorescence both in vitro (Figure S1A) and in vivo following 4 weeks or 6 months of treatment (Figure S1B). Importantly, immunofluorescence for Hevin revealed increased distribution in hippocampal astrocytes following 6 months of treatment with AAV‐GFAP‐Hevin when compared to controls (*p* < 0.05) (Figure S1C).

To evaluate cognitive decline during the progression of the disease, animals were housed for 6 months until they reached middle age (11–12 months old) and assessed in the following tasks: the novel object recognition (NOR), the Barnes Maze, and the novel object location (NOL) tasks. Importantly, littermate age‐matched WT animals were also measured in a blind fashion to be compared to APP/PSEN animals (Figure 2A). In this cohort, both middle‐aged WT and APP/PSEN mice injected with AAV‐GFAP‐Mock did not discriminate against novel objects following 24 h of object replacement (Figure 2B). Notably, middle‐aged APP/PSEN animals treated with AAV‐GFAP‐Hevin for 6 months normally discriminated between novel objects following 24 h of replacement (*p* < 0.01, as inferred by paired *t* Student's test), indicating that this treatment prevented memory decline in those animals (Figure 2B, Figure S2A). Moreover, AAV‐GFAP‐Hevin treatment also increased the interaction with novel objects in middle‐aged WT animals (*p* < 0.01, as inferred by paired *t* Student's test; treatment effect: *p* < 0.05, as inferred by two‐way ANOVA) (Figures 2A and S2A). Importantly, those animals were previously evaluated in this test during youth (5 months old) and displayed normal phenotype (*p* < 0.0001; *p* < 0.05, as inferred by paired *t* Student's test) (Figure S2B).

Next, to evaluate spatial memory acquisition, we used the Barnes Maze test during 5 consecutive days to measure the latency time to find the target hole. Middle‐aged APP/PSEN animals treated with AAV‐GFAP‐Mock presented decreased latencies until the third day of test (Figure 2C) when they reached a plateau with no further latency decrease. Middle‐aged APP/PSEN animals treated with AAV‐GFAP‐Hevin, on the other hand, displayed significant decreased latency to find the target starting in the third and fourth days of the test (*p* < 0.005, as indicated by multiple *t* test), indicating increased capacity to use spatial cues to favor spatial memory acquisition in comparison to control vector‐treated littermates (Figure 2C). Surprisingly, when evaluating middle‐aged WT animals treated with AAV‐GFAP‐Hevin, we also observed a significant reduction in the latency to find the target hole in the second and fourth days of test when compared to age‐matched animals treated with AAV‐Mock (*p* < 0.05, as indicated by multiple *t* test) (Figure 2D). When evaluating the performance of APP/PSEN and WT animals on the test day (day 5), we did not observe any significant difference in total latencies to find the target hole following Hevin overexpression (Figure S2C).

Finally, we evaluated animals using the NOL test, which is reported to reveal mid‐age cognitive decay in WT mice, as animals at this stage do not discriminate against novel located objects (Cabral‐Miranda et al. 2022). Remarkably, both middle‐aged WT and middle‐aged APP/PSEN animals treated with AAV‐GFAP‐Hevin showed increased interaction time with novel located objects as opposed to age‐matched littermates treated with AAV‐GFAP‐Mock (Treatment Effect: *p* < 0.0001; as indicated by two‐way ANOVA; *p* < 0.001; *p* < 0.05, as indicated by Tukey's multiple comparisons for WT and APP/PSEN, respectively) (Figures 2E and S2D).

**FIGURE 3:**
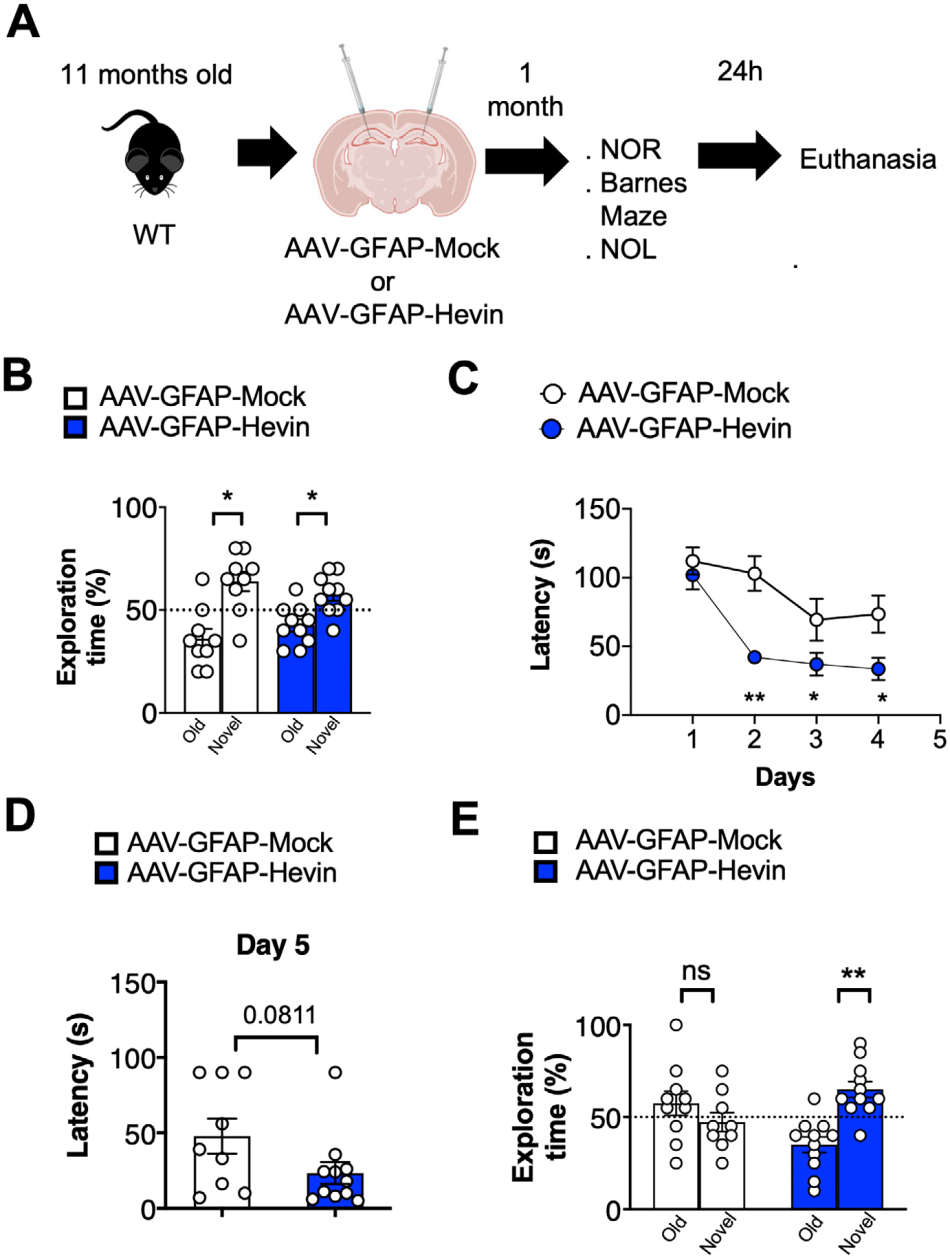
Gene delivery of Hevin in astrocytes through AAV prevents cognitive decline in middle‐aged WT and APP/PSEN animals following 1 month of treatment. (A) Workflow displaying genotypes, stereotaxic surgeries for AAV injection, behavioral tests, and end point analysis for AAV treatment for 1 month. (B) Percentage of exploration time of old or novel objects in the New Object Recognition task following 24 h of the training day. Middle‐aged WT type animals treated with AAV‐GFAP‐Mock or AAV‐GFAP‐Hevin for 1 month were evaluated in the test. *p < 0.05, Paired Student's *t* test. (C) Primary latencies to reach the target hole during 4 consecutive days in the Barnes Maze test. Middle‐aged WT animals treated with AAV‐GFAP‐GFP or AAV‐GFAP‐Hevin for 1 month were evaluated in the test. *p < 0.05; **p < 0.01, multiple *t* test. (D) Latency to find the target hole in test day (Day 5) of middle‐aged WT type treated with AAV‐GFAP‐Mock or AAV‐GFAP‐Hevin for 1 month. *p* = 0.081 as indicated by unpaired Student's *t* test. (E) Percentage of exploration time of old or novel located objects in the NOL task following 24 h of the training day. Middle‐aged WT animals treated with AAV‐GFAP‐Mock or AAV‐GFAP‐Hevin for 1 month were evaluated in the test. **p < 0.01, Paired Student's *t* test.

To further evaluate if a short‐term treatment through 1 month with AAV‐GFAP‐Hevin would render similar effects in WT animals, we performed the same battery of tests in another cohort of middle‐aged (11–12 months old) WT animals treated with either AAV‐GFAP‐Hevin or AAV‐GFAP‐Mock (Figure 3A). In this short‐term regimen, no differences were observed when comparing short term treatments in the NOR test, as this cohort of middle‐aged WT animals could discriminate novel objects following 24 h (*p* < 0.05, as inferred by paired Student's *t* test) (Figures 3B and S2E). Next, when employing the Barnes Maze test, we found that middle‐aged animals treated with AAV‐GFAP‐Hevin for only 1 month presented decreased latencies to find the target hole when compared to AAV‐Mock treated littermates starting at the second, third, and fourth day of the test (*p* < 0.01, *p* < 0.05; *p* < 0.05, as indicated by multiple *t* test) in the Barnes Maze (Figure 3C), and a trend of decreased latency was observed on the test day (Figure 3D; *p* = 0.081, as inferred by unpaired Student's *t* test). Finally, under this short‐term treatment with AAV‐GFAP‐Hevin, middle‐aged WT animals interacted significantly more with novel displaced objects in the NOL test (*p* < 0.01, as inferred by paired Student's *t* test) (Figures 3E and S2F).

Together, our findings suggest that boosting Hevin expression over a period of 6 months in hippocampal astrocytes effectively prevents cognitive decline in both normal and pathological aging. Additionally, a shorter, 1‐month increase in Hevin expression appears to provide cognitive benefits during the early stages of aging in middle‐aged mammals.

### 2.3 Hevin/SPARCL‐1 Overexpression in Hippocampal Astrocytes Does Not Impact Aβ Plaque Deposition in the Hippocampus

The presence of amyloid plaques is a hallmark histopathological feature of AD. These plaques consist mainly of aggregated beta‐amyloid (Aβ) peptides, which are derived from the amyloid precursor protein. The accumulation of Aβ peptides leads to the formation of insoluble fibrils, which deposit as plaques in the brain. These plaques are believed to contribute to the neurodegeneration seen in AD by inducing neuroinflammation, disrupting synaptic function, and promoting the aggregation of tau protein, another key pathological feature of AD (Jeremic, Jiménez‐Díaz, and Navarro‐López 2021). In that sense, therapies aimed at disrupting amyloid plaques have been a target for AD treatment.

**FIGURE 4:**
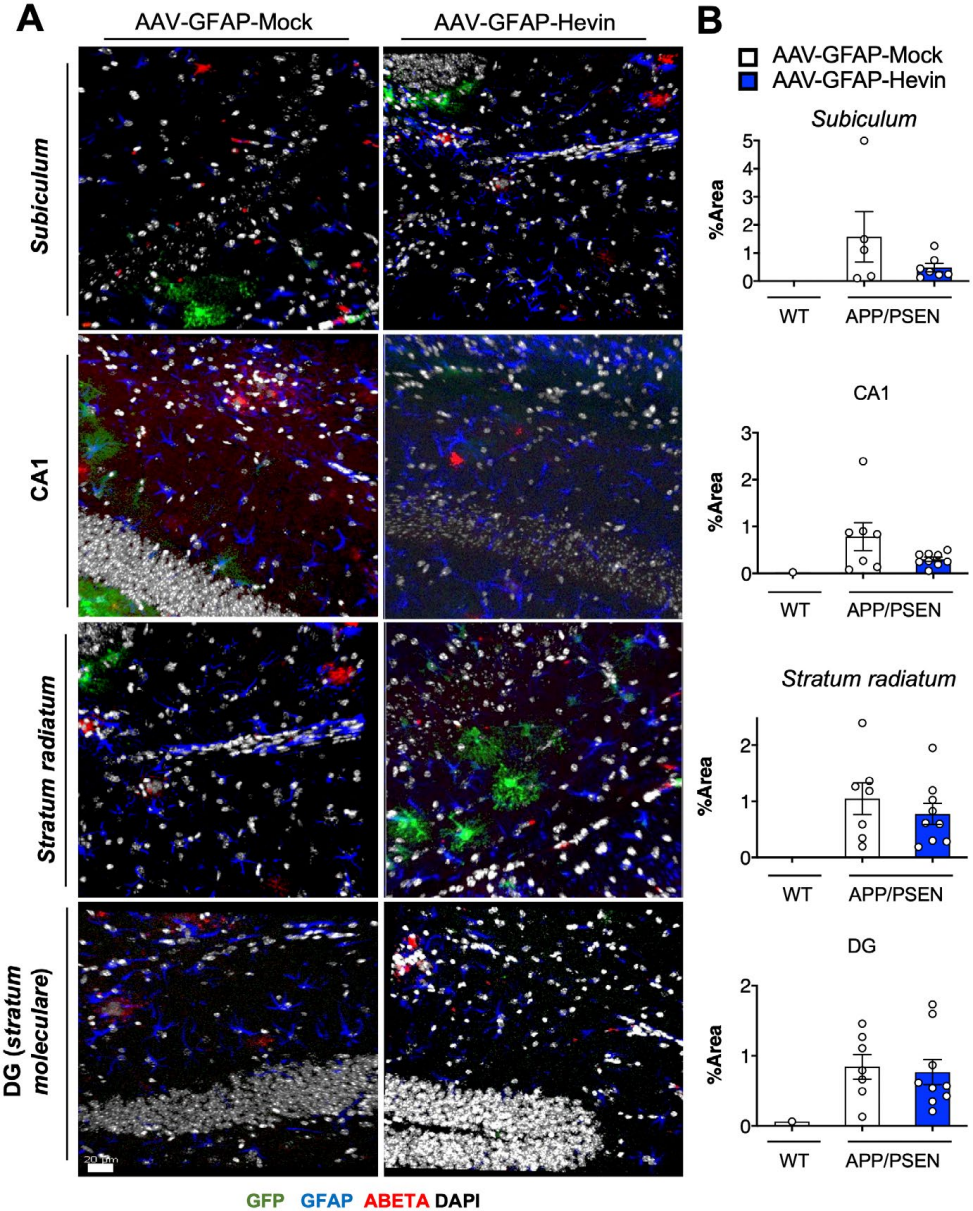
Astrocytic Hevin overexpression does not impact Aβ plaque deposition in the hippocampus of APP/PSEN animals. (A) Confocal microscopy acquired photomicrographs displaying immunofluorescence staining for GFP (green), Aβ plaques (red), and GFAP (blue) of different hippocampal regions (*subiculum*, CA1 region, *stratum radiatum*, and dentate gyrus) from middle‐aged APP/PSEN mice treated with AAV‐GFAP‐GFP or AAV‐GFAP‐Hevin. Nuclei are stained with DAPI (white). Magnification: 20×; scale bar = 20 μm. (B) Quantification of % area of Aβ immunolabeling in different hippocampal regions comparing middle‐aged APP/PSEN animals treated with AAV‐GFAP‐Mock or AAV‐GFAP‐Hevin. WT animals were used as a negative control for Aβ immunolabeling. Unpaired Student's *t* test, *p* > 0.05 for all comparisons.

To evaluate if Hevin overexpression could impact the deposition of amyloid plaques in the hippocampus of middle‐aged APP/PSEN mice, we used immunofluorescence and confocal microscopy to quantify Aβ plaques, in addition of GFAP and GFP, to localize astrocytes that have been transfected with AAV. Accordingly, we identified the deposition of Aβ plaques in the hippocampus subregions *subiculum*, CA1, *stratum radiatum*, and tangential zone of the dentate gyrus (DG) of middle‐aged APP/PSEN animals by immunofluorescence, which were absent in WT animals (Figure 4). Notably, our findings indicate that there was no significant alteration in the total area of immunolabeling against Aβ in neither of those subregions when comparing AAV‐Mock with AAV‐Hevin treated APP/PSEN animals, suggesting that our treatment did not significantly impact this pathological feature (*p* > 0.05; Figure 4).

Altogether, this data shows that Hevin overexpression in hippocampal astrocytes mitigate cognitive dysfunction in APP/PSEN animals by a mechanism independent of Aβ plaque formation/disruption.

### 2.4 Hevin Overexpression in Hippocampal Astrocytes Impacts the Composition of Synaptic Proteins and Increases Pre‐ and Postsynaptic Proteins Colocalization

**FIGURE 5:**
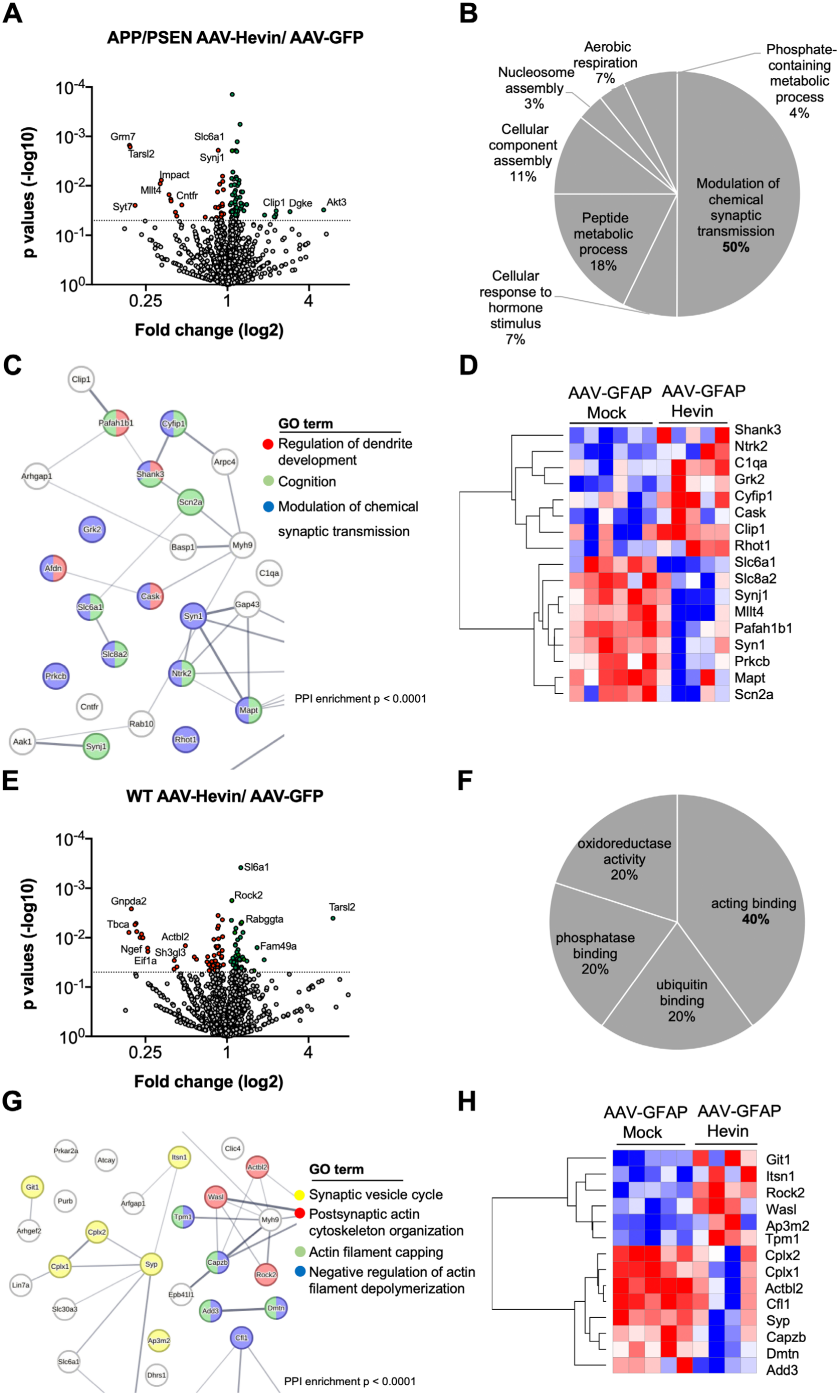
Astrocytic Hevin overexpression impacts the proteome composition of synaptic proteins both in middle‐aged WT and APP/PSEN animals. (A) Volcano plot displaying *p* values (−log_10_) and fold change (log_2_) of hippocampal proteomic alterations comparing middle‐aged APP/PSEN animals treated with AAV‐GFAP‐GFP or AAV‐GFAP‐Hevin. Cutt‐off is presented by the dashed line in *y* axis. (B) Pie chart presenting most significant enriched Gene Ontology terms of differentially expressed proteins comparing middle‐aged APP/PSEN animals treated with AAV‐GFAP‐GFP or AAV‐GFAP‐Hevin. (C) Zoom of a protein cluster in the protein–protein interaction network of differentially expressed proteins comparing middle‐aged APP/PSEN animals treated with AAV‐GFAP‐GFP or AAV‐GFAP‐Hevin (Gene Ontology terms: GO0050804: Modulation of chemical synapses (blue nodes), GO0050890: Cognition (green nodes) and GO0050773: Regulation of dendritic development (red nodes). Please refer to Data S1 for a full PPI network. (D) Heatmap comparing the expression (log_2_) of differentially expressed proteins related to Gene Ontologies discriminated in (C) in middle‐aged APP/PSEN animals treated with AAV‐GFAP‐GFP or AAV‐GFAP‐Hevin (*n* = 6, *n* = 5 biological replicates). (E) Volcano plot displaying *p* values (−log_10_) and fold change (log_2_) of hippocampal proteomic alterations comparing middle‐aged WT animals treated with AAV‐GFAP‐GFP or AAV‐GFAP‐Hevin. (F) Pie chart presenting most significant enriched Gene Ontology terms of differentially expressed proteins comparing middle‐aged WT animals treated with AAV‐GFAP‐GFP or AAV‐GFAP‐Hevin. (G) Zoom of a protein cluster in the protein–protein interaction network of differentially expressed proteins comparing middle‐aged WT animals treated with AAV‐GFAP‐GFP or AAV‐GFAP‐Hevin (Gene Ontology terms: GO009950: Synaptic vesicle cycle [yellow nodes], GO0098974: Postsynaptic Actin cytoskeleton organization [red nodes], GO0051693: Actin filament capping [green nodes], and GO0030835: Negative regulation of Actin filament depolymerization [blue nodes]). Please refer to Data S1 for a full PPI network. (H) Heatmap comparing the expression (log_2_) of differentially expressed proteins related to Gene Ontologies discriminated in (G) in middle‐aged WT animals treated with AAV‐GFAP‐GFP or AAV‐GFAP‐Hevin (*n* = 5, *n* = 4 biological replicates).

To further characterize the molecular mechanisms underlying the effects of astrocytic Hevin overexpression in age‐mediated cognitive decline, we performed proteomic evaluation of the hippocampus from APP/PSEN and WT animals treated with AAV‐GFAP‐Hevin or AAV‐GFAP‐Mock for 6 months (Figure 5A, Data S1). When comparing samples of APP/PSEN subjected to each treatment, we identified a total of 89 proteins differentially expressed by the two groups (Cut‐off: *p* < 0.05, *n* = 6, *n* = 5 biological replicates) (Figure S3A, Data S1). Gene set enrichment analysis using Gene Ontology for Biological Processes database revealed that 50% of the differentially expressed proteins were associated to biological terms related to the modulation of chemical synaptic transmission (Figures 5B and S3B), followed by a peptide metabolic process (18%) and cellular component assembly (11%). Protein–protein interaction network analysis indicated the formation of a significant network of proteins (PPI enrichment *p*‐value < 0.0001; Figure 5C; please refer to Data S1 for a complete PPI network) with a cluster displaying nodes related to modulation of chemical synaptic transmission (GO: 0050804), cognition (GO:0050890), and regulation of dendritic development (GO:0050773), which were associated to the following proteins: Ntrk2, Synj1, Slc6a1, Shank3, Slc8a2, Cyfip1, C1qa, Mapt, Afdn, Cask, Grk2, Pafah1b1, Rhot1, Syn1, Clip1, Prkcb, and Scn2a (Figure 5D); all of which are implicated in synaptic physiology.

Next, we compared the proteomic alterations of middle‐aged WT animals treated with AAV‐GFAF‐Mock or AAV‐GFAP‐Hevin (Figure 5E, Data S1) and found 103 proteins differently regulated in this condition (Cut‐off: *p* < 0.05; *n* = 5, *n* = 4, respectively) (Figure S3A, Data S1). Gene set enrichment analysis using Gene Ontology for Biological Processes indicated that 40% of enriched terms were associated to actin binding (40%), followed by ubiquitin binding (20%), phosphatase binding (20%), and oxidoreductase activity (20%) (Figures 5F and S3C). Protein–protein interaction network analysis highlighted a significant network of proteins (PPI enrichment *p* < 0.0001; Figure 5G; Data S1 for complete PPI network) with clusters displaying nodes related to GO terms: synaptic vesicle cycle (GO:0099504), postsynaptic actin cytoskeleton organization (GO:0098974), actin filament capping (GO:0051693), and negative regulation of actin filament depolymerization (GO:0030835), involving the following proteins: Syp, Cplx2, Cplx1, Git1, Itsn1, Ap3m2, Actbl2, Wasl, Rock2, Tpm1, Capzb, Add3, Dmtn, and Cfl1 (Figure 5H). This data indicates that in this condition, most protein clusters were connected to the dynamical processes mediating cytoskeleton polymerization and the secretion of neurotransmitters.

Notably, there was a minimal superposition of differentially regulated proteins comparing middle‐aged WT animals and middle‐aged APP/PSEN animals treated with AAV‐GFAP‐Hevin (Figure S3A, Data S1), as only 8 proteins (8.9% and 7.7%, respectively) were impacted in both conditions. Those findings suggest that despite Hevin overexpression presented a positive impact in both conditions (WT and APP/PSEN animals) as revealed by cognitive assessment, the mechanisms underlying these effects are likely to be different in respect to biological processes and molecular mediators implicated in synaptic function.

Finally, in order to evaluate if our findings were related to human AD pathology, we have compared a list of differentially expressed genes in astrocytes from patients (Sadick et al. 2022) with proteins differentially expressed in APP/PSEN mice treated with AAV‐Hevin (Figure S3D, Data S1). Interestingly, we identified 15 proteins altered in both comparisons (*p* < 0.001, Odds ratio: 15.78; as inferred by Fisher's exact test) including Ntrk2, Mapt, Slc6a1, and Atp9a, all of which are implicated in the modulation of chemical synapses and regulation of dendritic development (Figure S3D).

**FIGURE 6:**
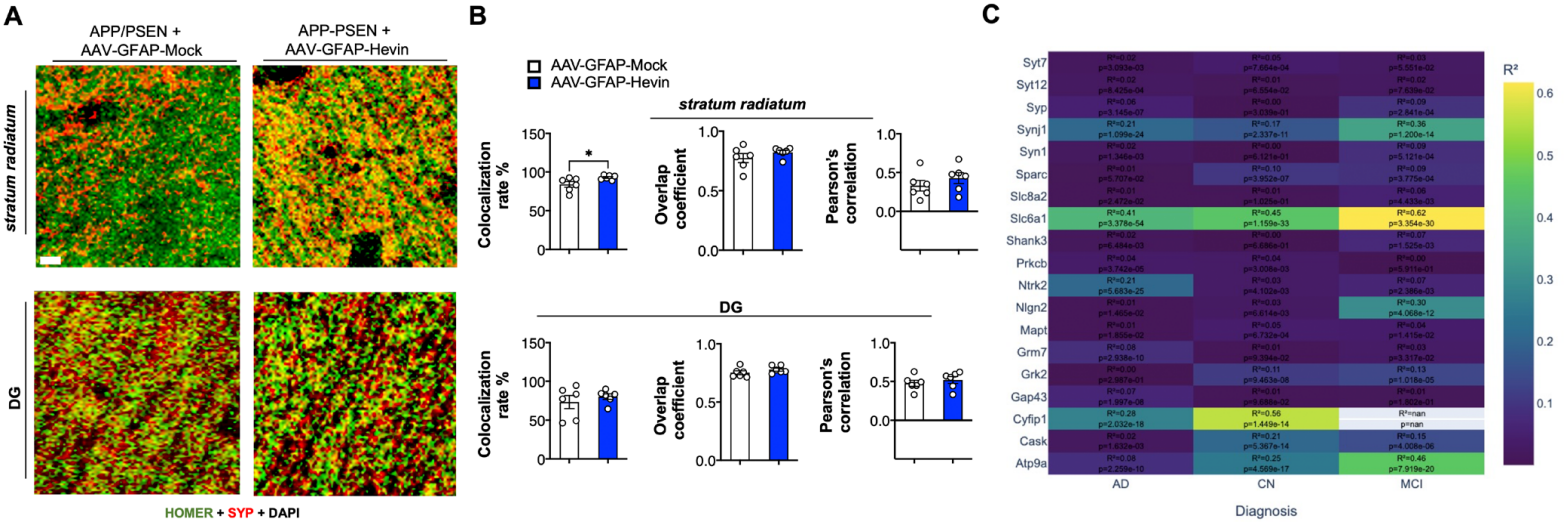
Astrocytic Hevin overexpression enhances synaptic colocalization in middle‐aged APP/PSEN mice and correlates with synaptic protein expression in human brain tissue. (A) Confocal microscopy acquired photomicrographs displaying immunofluorescence staining for presynaptic protein Synaptophysin (green) and postsynaptic Homer‐1 (red) of *stratum radiatum* and DG of middle‐aged APP/PSEN mice treated with AAV‐GFAP‐Mock or AAV‐GFAP‐Hevin. Magnification: 63X, scale bar = 5 μm. (B) Confocal microscopy‐based quantification of colocalization rate (%), overlap coefficient, and Pearson's correlation between green and red channels following image acquisition comparing *stratum radiatum* and DG from middle‐aged APP/PSEN mice treated with AAV‐GFAP‐Mock or AAV‐GFAP‐Hevin. *p < 0.05, unpaired Student's *t* test. (C) Regression analysis matrix displaying *r* ^2^ and *p* values comparing Hevin expression and the expression of a cluster of synaptic proteins in brain tissue of controls (CN), Alzheimer's disease patients (AD), and mild cognitive impaired (MCI) subjects. The cluster was identified by our proteomics analysis to be impacted by Hevin overexpression in the APP/PSEN animal model (Data S1). Colors indicate *r* ^2^ values.

Considering our findings in the proteomics evaluation and in behavioral assessments, we aimed to functionally evaluate whether astrocytic Hevin overexpression impacted the stabilization of synapses in the hippocampus of APP/PSEN mice. To do that, we used immunofluorescence and confocal microscopy to measure the colocalization of pre‐ and postsynaptic proteins in the *stratum radiatum* and tangential zone of the dentate gyrus of the hippocampus. We found that Hevin overexpression in APP/PSEN mice triggered increased colocalization of the presynaptic protein synaptophysin and the postsynaptic protein Homer‐1 in the *stratum radiatum* (*p* < 0.05, as indicated by unpaired Student's *t* test; Figure 6A,B) when compared to AAV‐Mock injected littermates, while no differences were observed in the tangential zone of the dentate gyrus (Figure 6C,D). As means to gain more insight into our data regarding fluorophore co‐occurrence and correlation, we have also evaluated Pearson's correlation and overlap coefficients but found no significant differences comparing treatments (Figure 6B,D).

In order to evaluate possible impact in synaptic physiology in WT animals, we have further evaluated middle‐aged WT animals treated with AAV‐GFAP‐Hevin or mock, but could not identify any significant differences in the colocalization rate in each treatment in both regions (Figure S4). Nonetheless, when evaluating the tangential zone of the DG, WT mice injected with AAV‐GFAP‐Hevin displayed increased Pearson's correlation and increased overlap coefficient when compared to AAV‐Mock treated littermates (Figure S4A), although no differences were observed in the colocalization rate (*p* > 0.05).

Finally, to study the contribution of Hevin in synaptic dysfunction in the physiopathology of AD, we have evaluated transcriptomics datasets arising from brain samples of AD patients, mild cognitive impaired (MCI) subjects, and age‐matched controls (GSE15222, GSE95587, and GSE125583). We have directed our analysis to the cluster of synaptic proteins that were identified to be impacted by Hevin overexpression following our proteomics assessment both in WT and APP/PSEN animals (Figures 5 and S3). Notably, linear regression analysis revealed that there was a significant positive association between Hevin levels and the expression of all the identified synaptic components (Figure 6C, Data S2), highlighting the expression of GABA transporter Slc6a1 (*r* ^2^ = 0.45 for controls; 0.41 for AD and 0,62 for MCI, *p* < 0.0001) and cytoskeletal dynamics regulator Cyfip1 (*r* ^2^ = 0.56 for controls and 0.28 for AD; Figure S4C).

Taken together, our findings indicate that astrocytic Hevin overexpression triggers proteomic alterations in the hippocampus of middle‐aged APP/PSEN and WT mice, impacting the proportion of colocalized pre‐ and postsynaptic proteins and Hevin levels are positively correlated with synaptic components expression in human brain tissue.

## 3 Discussion

Recent evidence supports that astrocytic Hevin/SPARCL‐1 exerts a central role in the cognitive impairment observed during normal aging, and in the course of AD pathogenesis (Strunz et al. 2019; Gan and Südhof 2019, 2020). When investigating this phenomenon using in vivo models of cognitive decline, we found that Hevin levels were decreased in hippocampal astrocytes when compared to WT animals, which was also observed in human astrocytes of AD patients compared to non‐demented elderly, as indicated by single cell RNAseq studies (Figure 1). We reasoned that by increasing Hevin levels in hippocampal astrocytes, we could abrogate the malfunctioning of aging synapses to ultimately ameliorate cognitive decline observed in AD pathogenesis. This hypothesis was confirmed by behavioral assessments and cellular and molecular analysis of synaptic proteins from AAV‐GFAP‐Hevin‐infected middle‐aged APP/PSEN animals.

Importantly, this was the first demonstration that increasing Hevin levels in hippocampal tissue could improve cognitive performance, as the Hevin role had been primarily described only in the context of neuronal development in specific circuits in the cerebral cortex and superior colliculus (Kucukdereli et al. 2011; Singh et al. 2016).

Our first hypothesis was directed to the evaluation of a mouse model of AD, namely, the APP/PSEN strain, which recapitulates several features of AD patients, including accelerated cognitive impairment, synaptic loss, and deposition of Aβ plaques in the brain (Sasaguri et al. 2017). Using this model, we reverted the cognitive decline observed in middle‐aged animals in addition to improving spatial memory acquisition, as indicated by distinct cognitive tests. Unexpectedly, we also observed improved cognitive outcomes in middle‐aged WT animals, which is in line with findings in humans, where *SPARCL‐1* mutations are associated with poor cognitive outcomes during aging and may potentially accelerate AD onset (Seddighi et al. 2017). Notably, previous studies indicated that *SPARCL‐1* mutations are associated with an increased risk of autism spectrum disorder (Taketomi et al. 2022), providing another piece of evidence that its regulation impacts the normal functioning of synapses.

One important finding elucidated by our proteomic assessment is that the clusters of differentially expressed proteins impacted by Hevin overexpression were substantially different in middle‐aged APP/PSEN and WT mice, with only 8 proteins being impacted in both conditions (Figure S3A). Importantly, despite those differences in individual protein expression, following gene set enrichment analysis, we determined that most enriched Gene Ontology terms were related to distinct aspects of synaptic physiology. In that sense, while in treated APP/PSEN animals, most significant terms were related to dendrite development and modulation of chemical synapses (Figure 5B–D), in WT animals, most terms were related to actin cytoskeleton physiology and synaptic vesicle cycle (Figure 5F–H). In the APP/PSEN background, regulated proteins related to synaptic plasticity included, among others, Shank3 and Cask, scaffold proteins that orchestrate dendritic spine and synapse formation and stabilization (Uchino and Waga 2013; Hsueh 2006), MAPT, a microtubule‐associated protein tau which is implicated in the physiopathology of tauopathies and other neurodegenerative diseases (Leveille, Ross, and Gan‐Or 2021), along with proteins Clip1 and Pafah1b1, also related to microtubule physiology (Hirokawa 1996; Moon and Wynshaw‐Boris 2013), and Ntrk2, a BDNF/NT‐3 growth factor receptor related to neuronal survival and synapse stabilization implicated in brain aging and AD (Levine et al. 1995; Colucci‐D'Amato, Speranza, and Volpicelli 2020). Interestingly, we also observed increased expression of the complement system mediator C1qa following Hevin overexpression, which is involved with inflammatory responses and is implicated in synaptic pruning by microglia and astrocytes interactions (Markarian et al. 2021; Färber et al. 2009; Cho 2019). When evaluating the behavior of those synaptic components in human datasets, we could determine that Hevin expression positively correlates with all of them, in line with our results, and protein Slc6a, a GABA transporter involved with intellectual disability and neurodevelopmental disorders (Trivisano et al. 2023), provided the strongest association.

When evaluating proteomic alterations exclusive to WT mice following Hevin overexpression, we detected altered expression of proteins related to synaptic vesicle cycle dynamics including synaptophysin (Syp), Cplx1 and 2, and Git1, in addition to regulators of actin polymerization including Tropomyosin alpha‐1 (Tpm1), Beta‐actin‐like protein 2 (Actbl2), Rho‐associated protein kinase 2 (Rock2), Cofilin‐1 (Cfl1), and Gamma‐adducin (Add3), among others (Figure 5G,H, Data S1). Interestingly, changes in the dynamics of actin filaments within dendritic spines and neuronal cytoskeleton are believed to stand as a central factor contributing to the early stages of brain aging (Mack, Kreis, and Eickholt 2016; Lai and Wong 2020; Kojima and Shirao 2007). Overall, our findings suggest that Hevin overexpression exerted important effects in regulating synaptic physiology by different mechanisms both in normal and pathological aging, converging in a similar outcome in preserving cognitive decline in both conditions. Those observations are in line with previous findings, indicating that astrocytic Hevin secretion stabilizes pre‐ and postsynaptic communication and synaptogenesis (Singh et al. 2016; Kucukdereli et al. 2011).

Here, we did not find significant differences in the deposition of Aβ plaques in the hippocampus of middle‐aged APP/PSEN mice following Hevin overexpression but found increased colocalization of pre‐ and postsynaptic proteins in the hippocampus. These findings suggest that the mechanism sustaining the Hevin‐induced improvements observed in cognitive tests were attributed to alterations in the composition of synaptic proteins and synaptic stability rather than disruption of Aβ plaques. Noteworthy, despite not impacting Aβ plaque deposition, APP/PSEN treated with AAV‐GFAP‐Hevin presented a significant improvement in cognitive performance when compared to age‐matched controls, highlighting an absent direct correlation between cognitive stability and Aβ deposition throughout pathogenesis. Importantly, despite evidence of Aβ neurotoxicity in vitro and in vivo animal models, studies in humans indicated that Aβ plaques and phospho tau neurofibrillary tangles are not exclusive to AD, and up to 30% of individuals in the normal aging population exhibit a similar amount of Aβ plaques in their brains as observed in typical AD cases (Iqbal, Liu, and Gong 2014; Gong, Liu, and Iqbal 2018).

The lack of success in clinical trials focused on Aβ as a therapy for AD has redirected attention toward alternative drug targets, in addition to highlighting the urgency in advocating for a paradigm shift in drug development for AD. In that sense, astrocytes emerge as a potential novel avenue of intervention in age‐mediated brain pathology, as those cells play a crucial role in the production of molecules essential for synaptic formation, maintenance, and plasticity including extracellular components such as thrombospondins, Hevin, SPARC, and glypicans; cholesterol, cytokines TNF‐α, TGF‐β1, and others (Diniz et al. 2012, 2014; Buosi et al. 2018; Allen and Eroglu 2017). Notably, deficits in astrocyte function and production of these molecules have been associated with synaptic dysfunction and cognitive decline observed in neurodegenerative diseases and in the aging brain (Pereira Diniz et al. 2017; Matias et al. 2022). Thus, understanding the milieu of synaptogenic molecules secreted by astrocytes subjected to the aging process and to AD pathogenesis may provide innovative avenues of treatment to ameliorate age‐associated cognitive decline (Fakhoury 2018).

In our model, we have evaluated synaptic integrity using an indirect methodology based on the colocalization of pre‐ and postsynaptic proteins through confocal microscopy, employing three distinct qualification methods for synaptic colocalization, including colocalization rates, overlap coefficients, and Pearson's correlation (Lagache et al. 2015; Aaron, Taylor, and Chew 2018), which encompass two separate aspects: fluorophore co‐occurrence and correlation. While co‐occurrence assesses the spatial overlap between two fluorophores, indicating the extent to which they coincide, correlation gauges the relationship between the abundance of two fluorophores that spatially overlap, focusing on functional or stoichiometric connections (Aaron, Taylor, and Chew 2018). There is evidence that APP/PSEN mice present decreased content of certain synaptic proteins (Almeida et al. 2005; Rutten et al. 2005), which was confirmed here by proteomics (Data S1); however, synaptic colocalization studies in this model present more complex results (Jordà‐Siquier et al. 2022; Martín‐Belmonte et al. 2020a, 2020b). Here, when examining the *stratum radiatum* region of the hippocampus of APP/PSEN mice treated with AAV‐GFAP‐Hevin, we found an increased colocalization rate of those markers, suggesting that our treatment improved the overlap of those particular synaptic components, in correlation with enhanced cognitive behavioral evaluation. No significant differences were found when analyzing overlap coefficients or Pearson's correlation in those animals. On the contrary, when analyzing the tangential zone of the DG of WT animals treated with AAV‐GFAP‐Hevin with controls, we observed a significant increase in both Pearson's correlation and overlap coefficients (*p* < 0.05, *p* < 0.01, respectively), indicating that our treatment also increased colocalization parameters in middle‐aged WT animals. Collectively, our data indicates that astrocytic Hevin overexpression differentially impacts the distribution of functional synapses in distinct regions of the hippocampus in normal and pathological aging. It remains to be addressed the impact of astrocyte heterogeneity in this event.

In this study, we observed that both long (6 months)‐ and short (1 month)‐term AAV‐GFAP‐Hevin treatments provided beneficial effects in age‐associated cognitive decline, sustaining that this molecule has the potential to positively impact aging synapses in different time windows even when age‐associated detrimental processes have already begun. Previous studies have shown that injection of blood from young into aged mice reversed age‐related cognitive impairments and enhanced synaptic connectivity in the brain, due to Hevin and thrombospondin‐4 (THBS4) enrichment in serum of young mice (Villeda et al. 2014; Gan and Südhof 2019). This effect was mainly due to enhanced dendritic arborization, synapse formation, and synaptic transmission (Gan and Südhof 2019).

In conclusion, we provide a proof of concept that by increasing astrocytic Hevin levels in the hippocampus, we abrogate cognitive decline both in pathological and normal aging highlighting Hevin as a potential novel intervention for age‐associated cognitive decline. In addition, Hevin is a secreted molecule which is present in human cerebrospinal fluid and serum (Nuñez‐delMoral et al. 2021), though its potential as a putative predictor biomarker for cognitive decline has not yet been investigated. Correlative studies of human cognitive decline and serum Hevin levels may provide evidence of Hevin potential as a biomarker for dementia. Overall, our findings provide evidence that astrocytic Hevin has the potential to revert age‐associated cognitive decline standing as a possible novel avenue of intervention for AD and dementia. Further, our research highlights the significance of targeting glial cells as potential targets for new therapies to reduce age‐related cognitive decline.

## 4 Materials and Methods

### 4.1 Animals

Animal handling and experimental procedures were previously approved by the Animal Use Ethics Committee of the Federal University of Rio de Janeiro (CEUA‐UFRJ, approved protocols: A23/21‐006‐18 and 122/22). Experiments were performed according to the Brazilian Guidelines on Care and Use of Animals for Scientific and Teaching Purposes (DBCA). Newborn (P0) C57BL/6N mice were used for astrocyte cultures. For in vivo experiments, adult male and female C57BL/6N and B6.Cg‐Tg (APPswe, PSEN1dE9) 85Dbo/Mmjax were used (4–12 months).

### 4.2 Astrocyte Cultures

Primary cultured astrocytes were derived from newborn C57BL/6N mice, as previously described (Matias et al. 2023). Briefly, cerebral cortices were removed, the meninges carefully stripped off, and hippocampi dissected. The tissues were maintained in Dulbecco's minimum essential medium (DMEM) and nutrient mixture F12 (DMEM/F12; Invitrogen), supplemented with 10% fetal bovine serum (FBS; Invitrogen). Cultures were incubated at 37°C in a humidified 5% CO_2_, 95% air chamber for approximately 7–10 days in vitro (DIV) until confluence was reached.

### 4.3 Adeno‐Associated Viruses (AAV) Injection

Young (6 months old) or middle‐aged (11 months old) C57BL/6N or APP/PSEN mice underwent single bilateral stereotaxic injections of AAV (AAV‐m‐SPARCL1/AAV‐272935, Vector Biolabs; 1 × 10^11^ viral genomes [VGs]/μL) in the hippocampi at coordinates antero‐posterior (AP): −1.9, dorso‐ventral (DV): +1.7, and medio‐lateral (ML): ±1.0. Mice were deeply anesthetized with 4% isoflurane, and a stereotaxic apparatus connected to a Hamilton microsyringe was employed for the procedure. The cranium was exposed through a skin incision, and bilaterally symmetrical holes were created using a dental drill. Injections were administered at a rate of approximately 0.5 μL/min for a total of 1 μL. After the procedure, mice were returned to their home cages and closely monitored until fully awake. The injections included either a control AAV serotype 2 vector (Mock) expressing eGFP, or AAV2‐GFAP‐Hevin, along with an eGFP cassette for transduction efficiency monitoring. AAV constructs included a GFAP promoter to drive transduction to astrocytes.

### 4.4 Behavioral Assessment Design

Behavioral experiments were performed in a blinded manner, both for genetically modified animals and AAV‐injected mice, using groups of age‐matched controls. Both males and females were injected and evaluated for all tests, but the majority of mice used in the study were female (70% for each group). Injected mice underwent behavioral evaluation 6 months or 1 month following AAV injections. Assessments were designed in line with bioethical guidelines restricting the total number of animals used in the study and were performed in the same order: NOR, Barnes Maze, and NOL.

#### 4.4.1 NOR

The NOR test, as previously described (Leger et al. 2013), consisted of a training phase followed by a test phase 24 h later. In the training phase, mice were placed in a round arena, initially facing away from two identical Lego blocks (3 × 4 cm) positioned distally. Mice were allowed to explore both objects until they accumulated a total of 20 s of interaction time, or up to 5 min if interactions were scarce. The test phase occurred 24 h after training, where one of the familiar objects from the training phase was replaced with a novel object, differing in color, shape, and texture. Mice were again allowed up to 20 s of exploration time (or 5 min if necessary). It should be noted that only one familiar and one novel object were presented at a time during each trial, with four different Lego blocks (3–4 × 4 × 4 cm) randomly assigned to trials across animals. Digital camera recordings were used to film the trials, and the arena and objects were cleaned with 70% ethanol between sessions to eliminate olfactory cues. Exploration time was defined as the duration the mice oriented their noses within 3 cm of an object, with behaviors like rearing or resting near the object excluded. Animals that failed to interact or exhibited distress were excluded from the analysis. Data analysis was conducted blind, and results were expressed as the percentage of time spent interacting with the novel object relative to the total interaction time. Additionally, to account for individual differences in exploration, the novelty index [(novel − familiar exploration time)/(novel + familiar exploration time)] was also calculated.

#### 4.4.2 Barnes Maze

The Barnes maze task utilized a white circular surface (0.9 m in diameter) with 20 evenly spaced holes around the perimeter, following established procedures (Cabral‐Miranda et al. 2022). An escape box (10 × 20 × 7.8 cm) was positioned beneath one hole, serving as the target location. A ramp facilitated mouse access to the escape tunnel. The elevated circular field had distal spatial cues (with various colors and shapes) placed outside the maze. A cylindrical start chamber was temporarily placed in the maze center and removed after 10 s. Training involved four daily trials for four consecutive days, each lasting a maximum of 3 min. If mice failed to find the target within 3 min, they were gently guided to the correct hole by the tail. A stopwatch sound served as an aversive stimulus to encourage exploration. Upon reaching the target, the noise ceased, and the mouse remained in the box for 1 min before returning to its home cage. A minimum 15‐min intertrial interval was observed for each animal. Ethanol cleaning between trials avoided olfactory cues. Primary pokes and primary latency to reach the target were manually recorded, referring to the initial pokes and time taken to reach the target for the first time, considering some animals did not promptly enter the escape box during training. Each trial was recorded on a video camera for subsequent analysis. On the fifth day, the escape box was removed for a single probe trial, evaluating spatial memory acquisition within a maximum time of 90 s, referred to as the test day.

#### 4.4.3 NOL

The NOL task was performed as previously described (Cabral‐Miranda et al. 2022) with small modifications. It was conducted using a squared arena divided into quadrants (15 × 15 cm^2^). In the training phase, mice were exposed to two identical Lego blocks (5 × 4 × 4 cm) placed in randomized quadrants, equidistant from each other. During the subsequent test phase, conducted 24 h later, mice were placed in the arena with one object moved to a different quadrant. Digital camera recordings were made for subsequent analysis of interaction time with the objects. Cleaning with 70% ethanol before each session prevented olfactory cues. Exploration time was defined as the duration mice oriented their noses toward an object within 3 cm. Following standard protocol, animals that failed to interact or exhibited signs of stress were excluded. Analyses were carried out in a masked fashion, and results were presented as the percentage of total time of interaction with old or novel objects in the test phase.

### 4.5 Immunofluorescence, Image Processing, and Quantification

Astrocyte cultures were fixed using paraformaldehyde at 4% in PBS and then washed and treated with a blocking solution (3% BSA and 0.05% Triton X‐100) for 60 min at room temperature. Brains were cut using a Vibratome (Leica, VT 1000Ssc514275) at 40 μm, and slices were collected in 24‐well plates with PBS. Slices were immersed in a blocking solution (3% BSA and 0.05% Triton X‐100) for 60 min at room temperature. The slices were then left to incubate overnight at 4°C with primary antibodies: anti‐GFAP (1:500, Invitrogen PA1‐10004 or 1:500, DAKO Z0334), anti‐beta‐amyloid (1:1000, Biolegend803002), anti‐GFP (Invitrogen A11122, 1:1000), anti‐Hevin (1:200, Santa Cruz sc‐514275), anti‐Homer (1:500, Abcam ab184955), or anti‐Synaptophysin (1:1000, MABN1193). The secondary antibodies used were anti‐chicken Alexa Fluor 633 (1:500, Invitrogen A‐21103), anti‐mouse Alexa Fluor 488 (1:200, Invitrogen A28175), anti‐rabbit Alexa Fluor 488 (1:200, Invitrogen A‐11008), and anti‐rabbit Alexa Fluor 546 (1:1000, Invitrogen A‐11035) in 3% BSA in TBS. Following three washes with PBS, DAPI was added in the final wash and incubated for 5 min. Imaging was performed using a Leica TCS SP5 confocal microscope with a 40× or 20× objective magnification.

An average of 2–3 images/animal/region was used for quantification in each experimental condition for quantification of AβO deposition (% labeled image area), synaptic puncta colocalization [including colocalization rate (%), Pearson's correlation, and overlap coefficients], or Hevin‐integrated density quantification (arbitrary units). LAS X software was used for inferring colocalization rate (%), Pearson's correlation, and overlap coefficients in colocalized images, all of which use different approaches for estimation (Adler and Parmryd 2010). Images were analyzed using the same parameters for each independent experiment. Image J was used to process confocal stacked images and quantify integrated densities and percentage areas of immunolabeled images.

### 4.6 Image Processing and Quantification

An average of 2–3 images per animal per brain region was acquired for quantification in each experimental condition. Quantifications included AβO deposition (% labeled image area), synaptic puncta colocalization metrics (colocalization rate, Pearson's correlation coefficient, and overlap coefficient), as well as Hevin integrated density quantification (expressed in arbitrary units). For quantification of integrated densities and percentage areas of immunolabeled images, ImageJ (FIJI) was used. Confocal stacked images were processed to retrieve maximal projections within each Z‐stack and highlight the brightest focal plane. Integrated density, defined as the product of area and mean fluorescence intensity, was calculated for each stacked image to quantify Hevin expression levels. To ensure accuracy, images were background‐subtracted and thresholded manually, maintaining the same threshold within each individual experiment. The percentage of the labeled image area for AβO deposition was determined using thresholding and particle analysis in ImageJ, allowing for precise quantification of immunolabeled regions.

For colocalization analysis, Leica LAS X software was employed to calculate the colocalization rate (%), Pearson's correlation coefficient (*r*), and the overlap coefficients (*K*_1_/*K*_2_), which assess the degree of overlap between two fluorescence channels, taking into account the intensity distribution of the signals. Pearson's correlation measures the linear relationship between pixel intensities in the two channels, while the overlap coefficients (*K*_1_/*K*_2_) consider both spatial overlap and signal intensity, offering a more nuanced interpretation of colocalization than traditional measures like the Manders' overlap coefficient (*M*_1_/*M*_2_) (Adler and Parmryd 2010). *K*_1_/*K*_2_ are weighted by the intensities and spatial distribution of overlapping pixels, providing insight into the proportion of one fluorophore's signal that overlaps with another. Each of these metrics provides complementary insights into the degree of colocalization, with consistent analysis parameters (e.g., thresholds and regions of interest) applied across all experimental conditions to ensure comparability.

All analyses were conducted using identical acquisition and processing settings across experimental conditions to minimize variability and enhance reproducibility. Confocal parameters (e.g., laser power, gain settings, and pinhole size) were optimized during the initial image acquisition and maintained consistently throughout the entire dataset collection. Quantification results were averaged across animals and regions and subjected to statistical analysis for comparison between experimental conditions.

### 4.7 Mass Spectrometry‐Based Proteomic Analysis for Animal Samples

A total of 4–6 animals per group was used for statistical analysis. Total protein extracts of hippocampus were prepared in TEN buffer (Tris‐HCl 10 mM pH 8.0; EDTA 1 mM; NaCl 100 mM) containing protease (mini cOmplete; Roche) and phosphatase (PhosSTOP; Roche) inhibitors cocktail using plastic pestles followed by ultrasonication (15 s; 20% power; QSonica). Then, the protein extracts were diluted to 1 μg/μL and 50 μg of protein per sample precipitated by adding 3 V of methanol, 1 V of chloroform, and 3 V of water (V = initial sample volume) followed by vigorous vortexing. The samples were centrifuged at 10,000 *g*, 1 min, 25°C to separate the precipitated protein in the interface between the aqueous and organic phases. The top phase (methanol/water) was removed without disturbing the protein layer and 5 V of methanol added to the sample, followed by vortexing and centrifugation at 10,000 *g*, 1 min, 25°C. The supernatant was removed, and the protein pellet washed with 5 V of methanol followed by vortexing and centrifugation as before. The protein pellet was air‐drying during 15 min at room temperature and solubilized in 1% solution of RapiGest SF (Waters), followed by thiol reduction with 5 mM of Tris (2‐carboxyethyl) phosphine hydrochloride for 30 min in 100 mM HEPES pH 8.0 at room temperature. Finally, thiol alkylation was performed with 10 mM iodoacetamide for 30 min at room temperature in the dark and proteins digested with 1 μg of trypsin (Promega) for 18 h at 37°C and 400 rpm vortexing. After digestion, peptides from 10 μg of proteins per sample were desalted using C18 Stage Tips (Rappsilber, Mann, and Ishihama 2007). Acetonitrile was evaporated on a SpeedVac. Peptide samples were resuspended in 100 μL of 0.1% formic acid, centrifuged at 18,000 *g* for 30 min to remove debris, and injected in the Orbitrap Fusion Lumos mass spectrometer coupled to a Nano EASY‐nLC 1200 (ThermoFisher). Peptides (from 100 ng of digested proteins) were first loaded in a trap column (nanoViper C18, 3 μm, 75 μm × 2 cm; ThermoFisher), then eluted onto a C18 column (nanoViper C18, 2 μm, 75 μm × 15 cm; ThermoFisher), and separated with a gradient of 5%–28% acetonitrile with 0.1% formic acid for 80 min, followed by 28%–40% acetonitrile with 0.1% formic acid for 10 min. The eluting peptides were detected in data‐dependent acquisition mode under positive electrospray ionization conditions. A full scan (*m*/*z* 400–1600) was acquired at a 120,000 resolution, followed by higher‐energy collisional dissociation fragmentation of the most intense ions in a 3 s cycle. Protein identification and label‐free quantification (LFQ) was performed using MaxQuant software, as described in de Melo et al. (2022). LFQ was compared between experimental groups and statistical analysis performed by multiple *t*‐test (Benjamini, Krieger, and Yekutieli 2006).

### 4.8 Pathway and Functional Protein Analysis

Differential protein expression lists were inputted in ClueGO application version 2.5.7 within Cytoscape (version 3.10.1). The functional analysis involved the Gene Ontology Biological Processes database with a medium network specificity chosen. Enriched biological pathways were displayed based on a threshold of *p* values < 0.05. Subsequently, these lists were input into STRING v. 12 to create protein–protein interaction networks, and the most representative gene ontology terms were color‐coded accordingly.

### 4.9 Statistical Analysis

Statistical tests used in this study included two‐tailed paired or unpaired Student's *t* test, two‐way ANOVA, followed by Tukey's multiple comparison post test, Wilcoxon rank‐sum test, Fisher's exact test, or Kolmogorov–Smirnov test depending on experimental evaluation and are indicated both in text and figure legends. Comparisons were considered statistical significance when *p* values were < 0.05. Regression analysis was performed using Python v. 3.9 in Anaconda Navigator 2.5.2 and libraries scikit‐learn v1.1.1, seaborn v0.11.2, pandas v2.2, and numpy v1.26.4. Regression models were run using read counts of synaptic proteins (reads per kilobase of transcript per million mapped reads) extracted from GSE 15222, GSE 95587, and GSE 125583 and Hevin read counts as a predictor. Associations were considered statistically significant when *p* < 0.05. *r* ^2^ values are also reported.

## Author Contributions

F.C.‐M., A.P.B.A., and F.C.A.G. conceptualization. F.C.‐M., A.B.P.A., and F.C.A.G. research design. F.C.‐M. and A.P.B.A. research performance. F.C.‐M., A.P.B.A., D.B.M., and F.C.A.G. data analysis. F.C.A.G. supervision. F.C.‐M. writing – original draft preparation. F.C.‐M. and F.C.A.G. reviewing and editing. D.M.S. and F.C.A.G. funding acquisition. F.C.A.G. project administration.

## Ethics Statement

Animal handling and experimental procedures were previously approved by the Animal Use Ethics Committee of the Federal University of Rio de Janeiro (CEUA‐UFRJ, approved protocols: A23/21‐006‐18 and 122/22). Experiments were performed according to the Brazilian Guidelines on Care and Use of Animals for Scientific and Teaching Purposes (DBCA).

## Consent

The authors have nothing to report.

## Conflicts of Interest

The authors declare no conflicts of interest.

## Supporting information


Data S1.

Data S2.

Figure S1.
Figure S2.
Figure S3.
Figure S4.

## Data Availability

All data are available in the main text or the [Link], [Link]. Proteomics raw data will be submitted in PRIDE depository.
