# Supplementary material for: Astrocytic Hevin/SPARCL‐1 Regulates Cognitive Decline in Pathological and Normal Brain Aging
Source: Aging Cell. 2025 Feb 12;24(5):e14493. doi: 10.1111/acel.14493 (PMC12074016; doi:10.1111/acel.14493)
Supplement: Supplementary file 3 — Figure S1. Figure S2. Figure S3. Figure S4. [file ACEL-24-e14493-s002.docx]

**Supplementary Material**

**Fig. S1.** Overexpression of Hevin in astrocytes delivered by AAV treatment *in vitro* and *in vivo.* A) Immunofluorescence for GFP of fixed cultured hippocampal astrocytes infected with AAV2-GFAP-Mock or AAV2-GFAP-Hevin for 48 hours. DAPI was used to label nuclei. 20X magnification. B) Immunofluorescence for GFP in the hippocampus of WT animals infected with AAV2-GFAP-Mock or AAV2-GFAP-Hevin for 4 weeks and 6 months. 40X magnification, scale bar = 20 μm. C) Left panels: Immunofluorescence for GFAP (green) and Hevin (red). DAPI was used to label nuclei. 40X magnification, scale bar = 20 μm. Right panel: Quantification of integrated density of the red channel, indicative of Hevin expression in astrocytes. Unpaired Student’s t test, p<0.05.

**Fig. S2. A)** Novelty index in the NOR test expressed by (novel-familiar exploration time)/(novel+familiar exploration time) of middle-aged WT and APP-PSEN animals treated with AAV-Mock or AAV-Hevin for 6 months. * p<0.05, as computed by unpaired Student’s t test. **B)** Percentage of exploration time of old or novel objects in the NOR task following 24 hours of the training day. Young WT or APP/PSEN animals were evaluated in the test. * p<0.01; **** p<0.001, Paired Student’s t test. **C)** Latencies to find the target hole in test day (Day 5) of middle-aged WT or APP/PSEN animals treated with AAV-GFAP-Mock or AAV-GFAP-Hevin for 6 months. p>0.05; as inferred by two-way ANOVA followed by Sidak multi-comparison. **D)** Novelty index in the NOL test of middle-aged WT and APP-PSEN animals treated with AAV-Mock or AAV-Hevin for 6 months. ** p<0.01, as computed by unpaired Student’s t test. **E)** Novelty index in the NOR test of middle-aged WT animals treated with AAV-Mock or AAV-Hevin for 1 month. p>0.05. **F)** Novelty index in the NOL test of middle-aged WT and APP-PSEN animals treated with AAV-Mock or AAV-Hevin for 1 month. * p<0.05, as computed by unpaired Student’s t test.

**Fig. S3. A)** Venn diagram displaying proteomic alterations of middle-aged APP/PSEN or middle-aged WT animals treated with AAV-GFAP-Mock or AAV-GFAP-Hevin. **B)** Gene set enrichment analysis displaying enriched Gene Ontology terms comparing differentially expressed proteins in the hippocampus of middle-aged APP/PSEN animals treated with AAV-GFAP-Mock or AAV-GFAP-Hevin (cut-off: p<0.05). Percentage of differentially expressed genes for each enriched term are shown. ** p<0.01. **C)** Gene set enrichment analysis displaying enriched Gene Ontology terms comparing differentially expressed proteins in the hippocampus of middle-aged WT animals treated with AAV-GFAP-Mock or AAV-GFAP-Hevin (cut-off: p<0.05). Percentage of differentially expressed genes for each enriched term are shown. ** p<0.01. **D)**  Venn diagram displaying proteomic alterations of middle-aged APP/PSEN treated with AAV-GFAP-Mock or AAV-GFAP-Hevin and transcriptome alterations in the astrocytes of AD patients compared to controls (as retrieved from (Sadick et al. 2022)).

**Fig. S4. A)** Confocal microscopy-based quantification of colocalization rate (%), overlap coefficient and Pearson’s correlation between green and red channels following image acquisition comparing tangential zone of the DG from middle-aged WT mice treated with AAV-GFAP-Mock or AAV-GFAP-Hevin. * p<0.05; ** p<0.01; unpaired Student's t test. **B)** Confocal microscopy-based quantification of colocalization rate (%), overlap coefficient and Pearson’s correlation between green and red channels following image acquisition comparing stratum radiatum from middle-aged WT mice treated with AAV-GFAP-Mock or AAV-GFAP-Hevin. p>0.05. **C)** Regression curves displaying the expression of the synaptic proteins Slc6a1 (up) and Cyfip1 (down) and Hevin in brain tissue of controls (CN), AD patients and mild cognitive impaired (MCI) subjects. R2 and p values are displayed, and diagnosis is indicated by colors. RPKM = Reads per kilobase of transcript per million mapped reads.

**Data S1. (separate file)**

Spreadsheet providing all detailed analysis performed for the MS-MS experiments.

**Data S2. (separate file)**

Spreadsheet providing all detailed analysis performed for regression analysis of human samples and the protein hits found in proteomics evaluation.
